# Adaptive Mechanisms of *Shewanella xiamenensis* DCB 2-1 Metallophilicity

**DOI:** 10.3390/toxics11040304

**Published:** 2023-03-25

**Authors:** Marina Abuladze, Nino Asatiani, Tamar Kartvelishvili, Danil Krivonos, Nadezhda Popova, Alexey Safonov, Nelly Sapojnikova, Nikita Yushin, Inga Zinicovscaia

**Affiliations:** 1 Andronikashvili Institute of Physics, I. Javakhishvili Tbilisi State University, 6 Tamarashvili Str., 0162 Tbilisi, Georgia; mabuladze46@gmail.com (M.A.); nina.asatiani@tsu.ge (N.A.); tamar.kartvelishvili@tsu.ge (T.K.); 2 Research Institute for Systems Biology and Medicine (RISBM), 18, Nauchniy Proezd, 117246 Moscow, Russia; 3 Department of Molecular and Translational Medicine, Moscow Institute of Physics and Technology, State University, 141700 Dolgoprudny, Russia; 4 Frumkin Institute of Physical Chemistry and Electrochemistry, Russian Academy of Sciences, 31, Leninsky Ave., 199071 Moscow, Russia; no.hope996@gmail.com (N.P.); alexeysafonof@gmail.com (A.S.); 5 Frank Laboratory of Neutron Physics, Joint Institute for Nuclear Research, 6 Joliot-Curie Str., 141980 Dubna, Russia; ynik_62@mail.ru (N.Y.); zinikovskaia@mail.ru (I.Z.); 6 Horia Hulubei National Institute for R&D in Physics and Nuclear Engineering, 30 Reactorului Str. MG-6, 077125 Bucharest, Romania; 7 The Institute of Chemistry, 3 Academiei Str., 2028 Chisinau, Moldova

**Keywords:** *Shewanella xiamenensis*, heavy metals, metal toxicity, antioxidants, genome sequencing

## Abstract

The dose-dependent effects of single metals (Zn, Ni, and Cu) and their combinations at steady time-actions on the cell viability of the bacteria *Shewanella xiamenensis* DCB 2-1, isolated from a radionuclide-contaminated area, have been estimated. The accumulation of metals by *Shewanella xiamenensis* DCB 2-1 in single and multi-metal systems was assessed using the inductively coupled plasma atomic emission spectroscopy. To estimate the response of the bacteria’s antioxidant defense system, doses of 20 and 50 mg/L of single studied metals and 20 mg/L of each metal in their combinations (non-toxic doses, determined by the colony-forming viability assay) were used. Emphasis was given to catalase and superoxide dismutase since they form the primary line of defense against heavy metal action and their regulatory circuit of activity is crucial. The effect of metal ions on total thiol content, an indicator of cellular redox homeostasis, in bacterial cells was evaluated. Genome sequencing of *Shewanella xiamenensis* DCB 2-1 reveals genes responsible for heavy metal tolerance and detoxification, thereby improving understanding of the potential of the bacterial strain for bioremediation.

## 1. Introduction

Environmental pollution by various toxic compounds is increasing every year. One of the most significant biosphere contamination problems worldwide is derived from heavy metals [1], which can be released by natural processes at relatively high levels. At the same time, anthropogenic activity associated with mining and different industrial activities, such as steel, leather, electroplating, mine tailings, paints, etc. [2], contributes significantly to the release of metal ions in the natural ecosystems.

Since the application of metals remains very high, it can have negative effects on human health. For example, lead, mercury, and copper adversely influence the nervous system, potentially leading to Alzheimer’s disease. Cadmium, mercury, and excessive amounts of zinc are particularly toxic to the kidneys and liver. Some metals (chromium, nickel, and lead) contribute to the development of malignant tumors. As a result, developing a strategy for polluted environment remediation is a matter of priority [3,4]. A set of physicochemical technologies is usually applied to remove heavy metals from the environment. However, all these methods have a series of shortcomings under natural environmental conditions [5].

The application of microorganisms from extreme habitats that possess resistance to xenobiotics is a promising approach for the development of modern waste management technologies. Bacteria, which form a major proportion of the earth’s biomass, have the greatest capacity to extract metals from the environment. Microorganisms’ resistance to heavy metal stress is manifested by their mobilization, sequestration, or transformation to fewer toxic forms [6]. Therefore, bacteria represent a vital concern for remediation processes.

All living organisms require some metals, known as trace elements or micronutrients, as basic building blocks while avoiding other toxic or potentially toxic elements. For example, metals like copper, zinc, iron, cobalt, chromium, and nickel in small amounts are necessary for biological growth and metabolism, but in larger concentrations, they can become toxic. It should be mentioned that for some elements, the range between concentrations inducing beneficial and hazardous effects is very narrow [7,8]. In bacteria, the resistance to physiologically required metals is ensured by an interrelationship between the resistance mechanism and the normal cellular metal metabolism. It allows the cell to accumulate the required metal for the physiological activities in sufficient amounts and to react to an increase in metal concentrations [9]. The intracellular concentrations of metals should be carefully controlled to avoid either metal deprivation or metal toxicity.

The main processes connected with metal toxicity in bacteria are the disruption of the bacterial cell membrane, the generation of reactive oxygen species (ROS), penetration through the bacterial cell membrane, and the induction of intracellular stress effects such as interaction with DNA and proteins [10,11,12]. Among the toxic effects provoked by heavy metals, the generation of ROS represents one of the major threats, resulting in disruption of fundamental metabolic processes and leading to cell death [11,12,13]. Control of ROS levels in bacterial cells is ensured by scavenging enzymes such as catalase (CAT), peroxidase (PER), and superoxide dismutase (SOD), which play critical roles in bacteria’s protection against oxidative stress [10,14,15].

Some bacteria (*Shewanella* spp., *Artrobacter* spp., *Bacillus* spp., etc.) from regions that are highly polluted with heavy metals reveal a special property, i.e., selective metallophilicity, mostly associated with the ability of microorganisms to change the degree of oxidation of heavy metal ions by transferring them into a safer, insoluble form and/or into complex compounds.

Under natural conditions, living organisms are rarely subjected to the action of only one chemical element; generally, organisms are exposed to polyelement pollutions [16]. Simultaneous exposure to heavy metals can cause toxic effects that are either additive, antagonistic, or synergistic [3].

The aim of the present study was to compare the effects of several heavy metals (Zn, Ni, and Cu) in single and multiple actions on the indigenous bacterium *Shewanella xiamenensis* DCB 2-1 (*S. xiamenensis* DCB 2-1) isolated from radionuclide-contaminated groundwater [17]. The choice of metals for study and the chemical composition of modeled systems were based on the chemical composition of real effluents from galvanic production [18]. Earlier in a series of works, the possibility of using *S. xiamenensis* DCB 2-1 biofilms as a biosorbent was investigated, and it was found that expanded clay and zeolite biofouling by *S. xiamenensis* significantly improved their sorption capacities toward nickel, zinc, and copper ions [19,20,21].

## 2. Materials and Methods

### 2.1. Chemicals

The chemicals used in experiments were purchased from Sigma Aldrich (Taufkirchen, Germany) and were of analytical grade.

### 2.2. Bacterial Culture and Growth Conditions

The bacterial culture of *S. xiamenensis* DCB 2-1 was isolated from a metal-contaminated environment [17]. The action of metals was studied using as a model the liquid cell culture at the stationary growth phase to exclude the probable influence of active proliferation processes on cell metabolism. Bacterial cells were aerobically grown in a culture medium with the following composition per 1 L: K_2_HPO_4_—2 g; FeSO_4_—10 mg; MgSO_4_·7H_2_O—200 mg; ammonium citrate monobasic C6H11NO7—1 g; yeast extract—1 g; glucose—1 g, at 30 °C with constant shaking. Culture growth was monitored by measuring optical density at 590 nm. Bacterial cells were grown for 24 h to obtain the stationary cell population, and then Zn(II), Ni(II), and Cu(II) ions as zinc chloride, copper chloride, and nickel chloride solutions in concentrations of 5, 20, 50, 75, 100, and 200 mg/L were added to the growth media. When adding the metal combinations Ni/Zn; Cu/Zn; Cu/Ni; and Cu/Ni/Zn, the single-metal concentration in each combination was 20, 50, 100, and 200 mg/L. The growth of cells in the presence of metal ions lasted 24 h. Cell viability was estimated by the colony-forming assay at the termination of the incubation time with single metals or their mixtures. The experimental conditions were identical for all studied cases.

### 2.3. Cell Viability

Cell viability in control and experimental variants was determined by the colony-forming assay (CFU). A cell suspension of 100 µL after several serial tenfold dilutions (from 10^−3^ to 10^−8^) was distributed on agar plates, followed later by the colony counting. The drop plate method was also used for the precise bacterial enumeration [22,23]. For the drop plate method, several dilutions have been exposed on one agar plate with 5–6 drops (10 µL) for each dilution. The drops were absorbed by agar in less than half an hour, and the plates were incubated in an inverted position. The number of colonies was counted on the third and fifth days after cell inoculation on agar plates. The total count of CFU for at least five drops at the countable dilution was averaged. The data are presented as the mean colony forming units per mL (CFU/mL) and as log CFU/mL, which are calculated by multiplying the number of colonies, the dilution factor, and the volume of the total sample.

### 2.4. Morphological Analysis

Morphological analysis of *S. xiamenensis* DCB 2-1 cells has been conducted by fluorescence microscope (LUMAM I-2, Leningrad, Russia) using intact acridine orange (AO) staining. AO (50 µg/mL) staining was performed on unfixed cells directly on microscope slides at room temperature for 5 min and then rapidly dried in the stream of warm air. Stained samples were visualized under an immersion objective.

### 2.5. Analytical Methods

For the detection of catalase, SOD activity, and total thiol content, cells were harvested by centrifugation at 10,000 rpm for 10 min (4 °C) and washed twice in 0.15 M NaCl. The cell pellets were treated with 500 µL of lysis buffer B-PER, pH 7.5 (Pierce, USA) per 2 × 10^9^ cells overnight at 4 °C. The lysate was centrifuged at 15,000 rpm for 20 min (4 °C). The supernatant was the crude cell extract. Protein concentration in the crude cell extract was determined by using BCA (bicinchoninic acid) protein assay reagent from Pierce (Rockford, IL, USA).

Sodium dodecyl sulfate polyacrylamide gel electrophoresis (SDS-PAGE) was performed by the method proposed by Laemmli [24], using 12.5% gels for 1 h at 30 mA. Twenty-five micrograms of the total protein were run per lane. The gels were stained with Coomassie blue R-250.

Native polyacrylamide gel electrophoresis was performed using 12.5% gels for 1 h at 30 mA. The crude cell extract was mixed with the loading buffer in the absence of 2-mercaptoethanol and SDS and loaded into the gel without heating. As the control, a crude cell extract from bacterial cells was used without metal addition.

### 2.6. Catalase Activity

Catalase activity in the crude cell extract was determined using a Catalase Activity Assay kit (Cayman Chemical, Ann Arbor, MI, USA) following the manufacturer’s instructions.

### 2.7. Superoxide Dismutase (SOD) Activity

The SOD activity was estimated qualitatively. The qualitative technique represented SOD in a gel assay. The method involves the photoreduction of nitro blue tetrazolium (NBT) for the determination of the activity of SOD following native polyacrylamide gel electrophoresis. The protein corresponding to SOD could then be visualized as an achromatic zone through the inhibition of NBT reduction via SOD. Total control loading was normalized by the equivalent protein quantity in the loading crude cell extract. Achromatic bands were visualized for 40 µg of protein equivalent. Following the electrophoresis, the gels were subjected to a two-step staining procedure [25].

### 2.8. Total Thiol Content

The procedure of total thiol (SH-group)-containing compound detection in the crude cell extract is based on the use of 5,5′-dithiobis-(2-nitrobenzoic acid) (DTNB), which at interaction with SH groups produces a chromogenic substrate [26]. The range of GSH (glutathione reduced form) concentrations of 0–0.5 mM served as the standards. The developed color was read at 412 nm.

### 2.9. Determination of Metal Content

For the measurement of metal uptake, the biomass was separated from the medium by filtering it through a membrane with a pore diameter of 0.22 µm manufactured by Vladipor (Vladimir, Russia) on a vacuum unit, and aliquots of the filtrate were used to analyze the residual content of metals. The concentrations of metals in the control and experimental solutions were determined by an ICP-OES PlasmaQuant PQ 9000 Elite spectrometer (Analytik Jena, Jena, Germany). The calibration solutions and standards were prepared from IV-STOCK-27 (Inorganic Ventures, Christiansburg, VA, USA) standard solution. All control standards were analyzed after every 10 samples.

The content of metal accumulated by biomass was calculated using the following equation:

(1) $$q=\frac{V(C_{i}-C_{f})}{m}$$

where V is the volume of the solution, in mL; Ci and Cf are the initial and final metal concentrations, in mg/L; and m is the amount of biomass, in g.

### 2.10. Genome Analysis

The primary annotation of the genome was carried out using the PROKKA program and refined using the annotation obtained by BLAST KOALA. The role of some proteins was separately clarified by BLASTp (base: non redundant protein). The search for genes responsible for metal resistance was implemented by mapping protein sequences to the BacMet experimental database (e-value < 1 × 10^−25^, Per.Identity > 30). Subsequently, the mapped sequences were verified using literature data.

## 3. Results

### 3.1. Biological Response to Single and Multiple Metals Action

**Figure 1:**
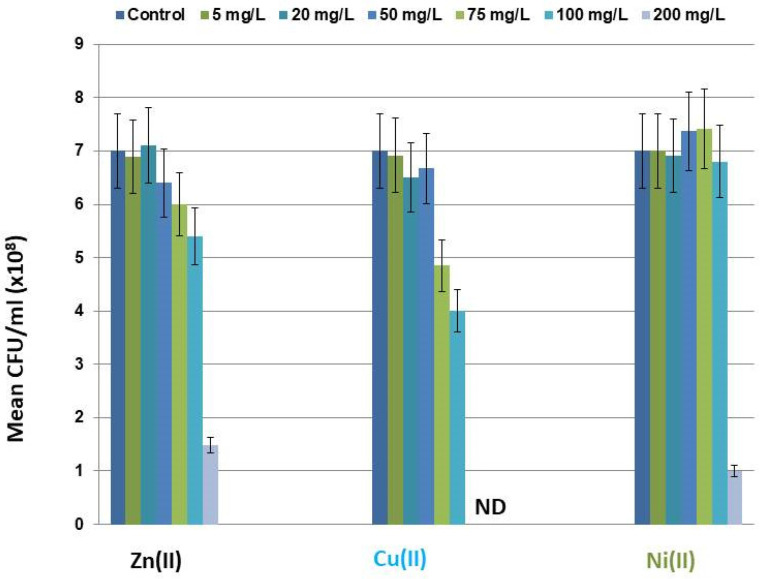
The dose-dependent impact of zinc, copper, and nickel on the *S. xiamenensis* DCB2-1 cell population is presented as CFU/mL. The data presented are the mean values ± SD from three separate sets of experiments.

**Figure 2:**
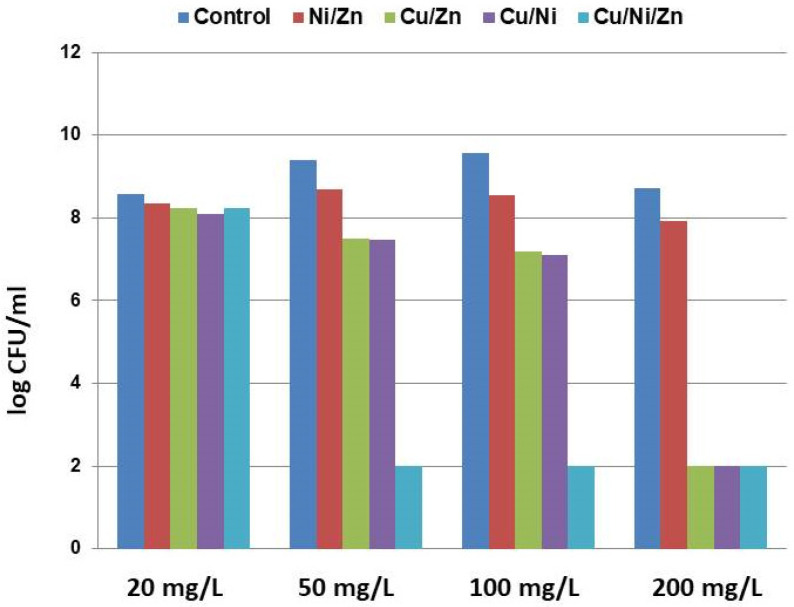
The dose-dependent impact of zinc, copper, and nickel combinations on *S. xiamenensis* DCB2-1 cell population is presented as log (CFU/mL).

The cytotoxicity induced by Zn(II), Ni(II), and Cu(II) (Figure 1) and their mixtures Ni/Zn, Cu/Zn, Cu/Ni, and Cu/Ni/Zn (Figure 2) in the batch culture of *S. xiamenensis* at the stationary growth phase exhibited different characteristics depending on the increasing metal concentrations in the medium.

The bacterial cell culture was treated with Zn(II), Ni(II), and Cu(II) in the concentration range of 5–200 mg/L. The addition of Zn(II) to the growth media at the studied concentrations caused a mild dose-dependent toxic effect on the cell population (Figure 1), leading to a gradual decrease in the cell number compared with the untreated control (7.1 × 10^8^ CFU/mL) at Zn(II) concentrations of 75 mg/L (6 × 10^8^ CFU/mL), 100 mg/L (5.0 × 10^8^ CFU/mL), and 200 mg/L (1.4 × 10^8^ CFU/mL). The reduction of the cell population was less than one order of magnitude for all studied concentrations. At the highest applied concentration of 200 mg/L, the number of live cells prevailed in the population. The microscopic fluorescent analysis using the AO stain confirmed that the majority of cells in the bacterial population were viable and metabolically active.

The cell culture of *S. xiamenensis* was tolerant to the presence of Ni(II) in the growth media for 24 h in the concentration range of 5–100 mg/L. The bacterial cells viability was more stable under the action of Ni(II) at the tested concentrations than under Zn(II) action. The decrease in cell number in the population was observed only at Ni(II) concentrations of 200 mg/L (Figure 1), and, as in the case of Zn(II), it did not exceed one order of magnitude.

Cu(II) action was characterized by the linearity of the dose-response effect (Figure 1). The toxic action was detected at a Cu(II) concentration of 75 mg/L (4.85 × 10^8^ CFU/mL) and enhanced at 100 mg/L (4.0 × 10^8^ CFU/mL) (compare to the untreated cell culture (7.1 × 10^8^ CFU/mL)). The further increase in the metal concentration up to 200 mg/L caused the cell-destructive processes. Colonies were not detected at a Cu(II) concentration of 200 mg/L. Moreover, microscopic fluorescent analysis showed practically no live cells in this case. Therefore, among the studied metals, only Cu(II) generated the dose-dependent severe toxicity resulting in the annihilation of live cells in the population. The Cu(II) concentrations of 75 and 100 mg/L could be considered sub-toxic.

The cytotoxicity induced by the mixtures of metals Zn(II), Cu(II), and Ni(II) at administration in combinations into the growth media was assessed as the logarithm of the average number of colonies (CFU/mL) in Figure 2. Within the studied metal combinations, the mixture of Ni/Zn was characterized by the lowest toxicity; the number of cells decreased by an order of magnitude only at high concentrations (50, 100, and 200 mg/L) for each metal. The cell population of *S. xiamenensis* DCB2-1 mostly consisted of viable cells, but the viability was lowered compared to the single action of Ni(II) or Zn(II). The decline of viable cells was not detected at the concentration of 20 mg/L.

As a result of exposure to Cu/Zn and Cu/Ni combinations, the number of cells decreased by two orders of magnitude at metal concentrations of 50 and 100 mg/L, and the live cells were not detected at 200 mg/L. At the multiple action of Cu in combination with Zn or Ni at a sub-toxic concentration of 50 mg/L, the number of cells declined considerably in comparison with a single Cu(II) system.

The effects of combinations of the studied metals differed by the rate of their dose-dependent toxicity. The sub-toxic effect on the cell population in the Ni/Zn system was observed at a concentration of 200 mg/L. The Cu/Ni and Cu/Zn systems resembled each other in their actions on the cell culture of *S. xiamenensis* DCB2-1, producing the sub-toxic effect at metal concentrations of 50 and 100 mg/L and generating the toxic action at a concentration of 200 mg/L. As for the Cu/Ni/Zn combination, the concentrations of 50, 100, and 200 mg/L caused severe toxicity, and live cells were not detected after 24 h of metal administration into the growth media, neither by the colony-forming assay nor by microscopic fluorescent analysis.

It has been demonstrated that the combination of the above-mentioned metals at a concentration of 20 mg/L of each metal was non-toxic for all studied multi-metal systems: Ni/Zn, Cu/Zn, Cu/Ni, and Cu/Ni/Zn.

### 3.2. The Accumulating Characteristics of Single and Multiple Metals by S. xiamenensis

Metal-accumulating characteristics of bacterial cells reflect two processes: adsorption onto the bacterial surface and intracellular accumulation. Adsorption is a rapid process, while accumulation is slow and energy-dependent. Both processes take place in live cells under non-toxic and sub-toxic metal actions. The metabolism-independent adsorption occurs mostly in dead cells under toxic metal action. The assessment of the single and multiple metal accumulation by *S. xiamenensis* DCB2-1 is presented in Table 1 and Table 2, respectively.

An increase in zinc concentration in the cultivation medium resulted in an increase in its content in bacterial biomass (Table 1). Thus, at Zn(II) concentration in solution of 5 mg/L, its content in biomass constituted 6.23 mg/g and increased up to 288 mg/g at metal concentration of 200 mg/L. Starting with a concentration of 20 mg/L, Zn(II) was almost completely removed from the medium (90–96%).

The accumulation of copper by biomass was less pronounced when compared with Zn(II); however, the straight-line correlation between metal content in solution and biomass was maintained (Table 1). With the increase of Cu(II) concentration in solution from 5 to 100 mg/L, its content in biomass varied between 2.3 and 38.2 mg/g, and the efficiency of uptake was ranging from 6 to 19%.

Among the studied metals, *S. xiamenensis* DCB2-1 showed the lowest uptake in the case of Ni(II) (Table 1). The highest uptake of 30 mg/g was achieved at a Ni(II) concentration in solution of 200 mg/L. The efficiency of Ni(II) uptake from solution was less than 21%, and it decreased with the rise of Ni(II) concentration in solution. Therefore, low Ni(II) toxicity is correlated with low uptake by *S. xiamenensis* DCB2-1.

Regarding metal uptake in bacterial biomass grown in multi-metal systems, the efficiency of metal uptake differed from single-metal systems. Thus, in the Cu/Ni system at a metal concentration of 20 mg/L, the efficiency of copper uptake increased 50 times compared to the Cu(II) system, and that of nickel increased 6.3 times compared to the single-metal system. In the Ni/Zn system, at the same metal concentrations (20 mg/L), the increase in nickel content in biomass was comparable with the Cu/Ni system, while zinc content was on the level of a single system. In the Cu/Zn system, copper uptake was 43 times higher than in the Cu(II) system, while the content of zinc decreased compared to the Zn(II) system (by 33%). In the three-metal system, the same tendency was observed: the content of copper and nickel in the biomass increased 55 and 4.3 times, respectively, in comparison with single-metal systems, while the content of zinc similarly decreased (by 33%).

At metal concentrations of 50 mg/L in the Cu/Ni system, the content of both elements was higher than in single-element systems, 22 times for Cu(II) and 7.2 times for Ni(II). In the Cu/Zn system, the content of copper accumulated by biomass was 13 times higher in comparison with the single-metal system, while the accumulation of zinc was 91% lower. The uptake of elements in the Ni/Zn system was similar to the same system at metal concentrations of 20 mg/L. It should be mentioned that a concentration of 50 mg/L for each metal is toxic for the combination Cu/Ni/Zn; at the same time, this concentration is non-toxic for single-metal reactions. In this case, it was not possible to compare the efficiency of uptake between multi-metal and single-metal systems because bacterial populations were in different biological states. However, the process of removal of metals from the solution by the dead bacterial cells in the Cu/Ni/Zn solution took place, and the efficiency of metal removal was comparable: 16.1% for copper, 12.6% for nickel, and 10.7% for zinc (Table 2).

The same pattern was obtained for a metal concentration of 200 mg/L (Table 2). The bacterial population at 200 mg/L in the combinations Cu/Ni, Cu/Zn, and Cu/Ni/Zn consists of dead cells. The efficiency of metal removal by the bacterial cells from Cu/Ni solutions is 43.2% for copper and 21.8% for nickel. The efficiency of metal removal from Cu/Zn solutions was comparable for copper (14.9%) and zinc (19.6%) systems. Bacterial cells removed the metals from the Cu/Ni/Zn solution in the following order: zinc (78.6%) > copper (28%) > nickel (21.3%).

The exception in multi-metal systems at a concentration of 200 mg/L was the combination Ni/Zn. According to the viability data, this concentration was sub-toxic for Ni(II) and Zn(II) single systems, as well as the Ni/Zn combination. In this case, Zn(II) uptake from the multiple system was comparable with the single-metal system, but Ni(II) uptake in the multiple system increased three times in comparison with the single system. The uptake of Ni(II) in all multiple systems was higher than in Ni(II) single systems.

### 3.3. Gene Analysis

As a result of the mapping of protein sequences to the BacMet experimental database, 120 genes associated with metal resistance were identified. By comparing the results of the BLAST KOALA and BLASTp annotations and using literature data, it was determined that 85 genes are most likely to play a role in metal resistance (Supplementary Materials).

**Figure 3:**
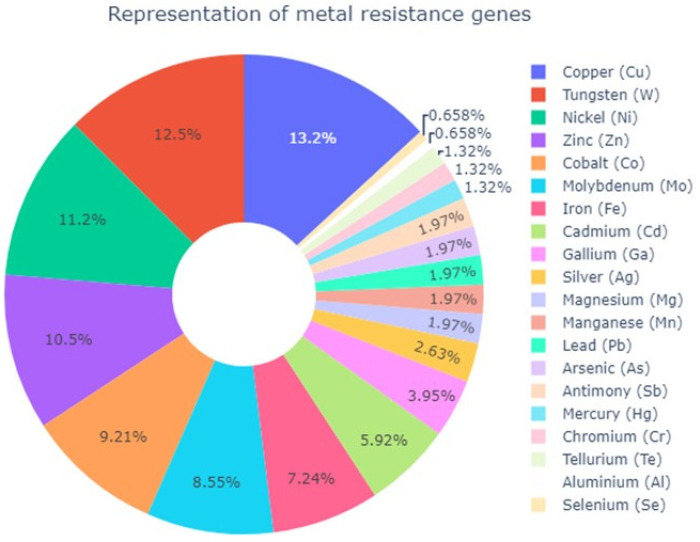
The number of genes associated with metal resistance in *S. xiamenensis* DCB2-1.

It was found that the main parts of genes, identified as genes associated with metal resistance, were resistant to copper (Figure 3). This is due to a significant representation of the genes encoding the sigma-54-dependent regulator of the DNA binding response (ONJFFADL_00184, ONJFFADL_00630, ONJFFADL_00632, ONJFFADL_00999, ONJFFADL_01236, ONJFFADL_01236, ONJFFADL_01468, ONJFFADL_01562, ONJFFADL_01655, and ONJFFADL_03365) [27]. This regulator reacts to copper and induces the production of carotenoids, while it regulates two P-type ATPases: copA (ONJFFADL_02161) and copB (ONJFFADL_02196) [28]. In addition, the bifunctional protein disulfide isomerase/oxidoreductase DsbC (ONJFFADL_00291) was found in the genome and is involved in the protection of cells from oxidative stress of the membrane and copper stress [29]. The discovered CueR (ONJFFADL_02162) controls the transcription of two copper homeostasis genes: copA (ONJFFADL_02161), encoding the P-type ATPase Cu (II)—transport pump, and cueO (not identified), encoding copper oxidase for detoxification [30].

Cu(I)-sensitive transcription regulators (ONJFFADL_02162 and ONJFFADL_02459) are responsible for activation of the copper efflux system [31,32]. ONJFFADL_00967 represents the copper regulator, CusR [33]. ONJFFADL_02433 has been annotated as the cusB subunit of the periplasmic outflow adapter RND and is associated with the transport of copper and silver ions [34]. It is noted that cusA, cusB, copA, and copB are also involved in the formation of resistance to silver.

Zinc transfer is realized using the ZntR transporter (ONJFFADL_00816) [35]. Furthermore, it is assumed that zinc resistance is formed through some transcription factors (ONJFFADL_01264 and ONJFFADL_03080) [36]. In addition, the genome contains two proteins that provide resistance to cobalt, zinc, and cadmium, CzcA and CzcB, which regulate the concentration of Cd, Zn, and Co (ONJFFADL_02598 and ONJFFADL_02597) [37]. Three proteins have similar CzcA and CzcB functions (ONJFFADL_03517, ONJFFADL_03517, and ONJFFADL_01547). Zinc content is also regulated by a number of other proteins listed in Supplementary Materials.

The level of Ni(II) ions is regulated by ATP-dependent transporters (ONJFFADL_03077, ONJFFADL_03076, and ONJFFADL_01609). The ONJFFADL_03983 protein belongs to the HupE/UreJ family, which is involved in nickel transfer [38]. In addition, a TonB-dependent siderophore receptor (ONJFFADL_01301) was discovered and shown to be able to resist stress caused by metal ions such as Ni(II), Cu(II), and Pb(II) [39]. Furthermore, Ni/Co carriers ONJFFADL_01210, ONJFFADL_01211, ONJFFADL_01252, ONJFFADL_01987, and ONJFFADL_02147 [40] and carriers of several metals ONJFFADL_03517, ONJFFADL_02543, and ONJFFADL_00995 were identified.

### 3.4. Catalase Activity in Response to Single and Multiple Metals Action

Further, the dose-dependent impacts of single metals and their combinations on *S. xiamenensis* stress adaptation for the single metals at the non-toxic concentrations of 20 and 50 mg/L and their combinations at 20 mg/L of each of the metals have been estimated. The metabolically active cells have been employed for the estimation of antioxidant system status. The bacteria were cultivated for 24 h before the metal addition to achieve substantial enzyme production at the stationary growth stage.

**Figure 4:**
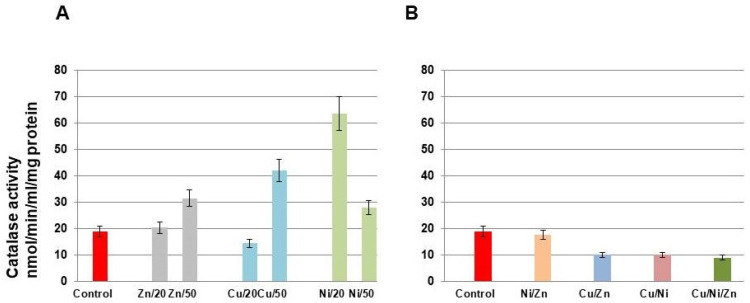
Catalase activity in the cell lysate of *S. xiamenensis* DCB2-1 under zinc, copper, and nickel single (**A**) and combination actions (**B**). Metal concentrations in single systems are 20 and 50 mg/L; metal concentrations in complex systems are 20 mg/L. The data presented are the mean values ± SD from three separate sets of experiments.

Figure 4 represents the activity of catalase at different concentrations of individual metals and their combinations. According to Figure 4A, the activity of catalase under Ni(II) action increased significantly at a metal concentration of 20 mg/L compared to the control. As concerns Cu(II), the activity of catalase decreased at 20 mg/L, while Zn(II) at the given concentration did not affect the catalase activity. The activity of catalase at a metal concentration of 50 mg/L increased in comparison with the control activity, most significantly under Cu(II) action. Based on the data, catalase, as a member of the oxidative stress protectors, is very effective in response to Ni(II) action at both studied concentrations (20 mg/L and 50 mg/L) and actively defends the cells from Cu(II) action at a concentration of 50 mg/L in a single-metal system. In the case of joint action of metals, only the combination Ni/Zn revealed activity comparable with the control; in all other cases, the activity of catalase was inhibited (Figure 4B).

### 3.5. SOD Activity in Response to Single and Multiple Metals Action

**Figure 5:**
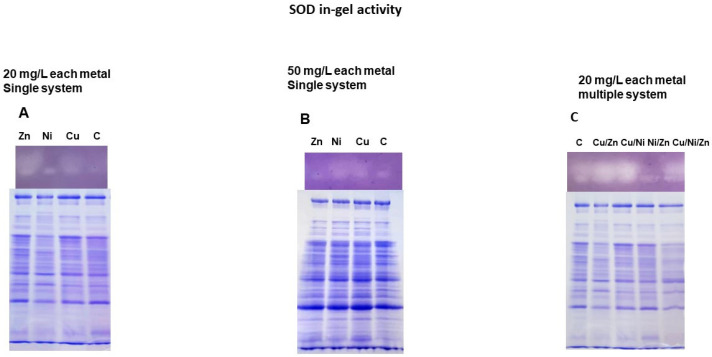
SOD activity in the cell lysate of *S. xiamenensis* DCB2-1 under zinc, copper, and nickel single (**A**,**B**) and combination actions (**C**). Metal concentrations in single systems are 20 and 50 mg/L; metal concentrations in complex systems are 20 mg/L. C—Control sample.

The SOD activity was qualitatively estimated using the SOD-in-gel assay technique. Figure 5 shows SOD activity in response to the action of single metals and their combinations. Figure 5 contains the conventional protein electrophoresis data, which are loading controls in the sense that the same amount of protein was applied. When cells have been growing in the presence of 20 mg/L of each metal, increased SOD activity was observed under Zn(II) and Cu(II) actions (Figure 5A). An increase in the concentration (50 mg/L) for Ni(II) and Cu(II) is accompanied by an increase in SOD activity (Figure 5B). With regards to the joint action of metals, an increase in SOD activity was observed for all combinations except Ni/Zn; this combination caused a decrease in SOD activity in comparison with the control (Figure 5C).

### 3.6. Total Thiol Content in Response to Single and Multiple Metals Action

**Figure 6:**
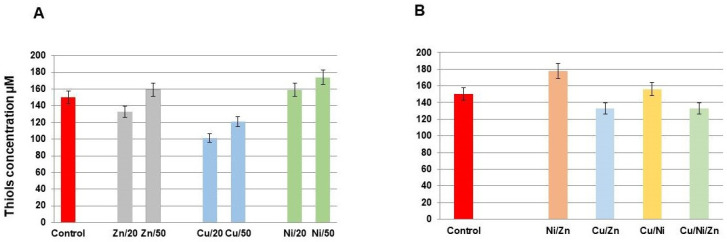
Total thiol content in the cell lysate of *S. xiamenensis* DCB2-1 under zinc, copper, and nickel single (**A**) and combination action (**B**). Metal concentrations in single systems are 20 and 50 mg/L; metal concentrations in complex systems are 20 mg/L. The data presented are the mean values ± SD from three separate sets of experiments.

Figure 6 represents the total thiol content in bacterial cells at different concentrations of metals in single and multiple systems. According to Figure 6A, under the action of 20 mg/L of each metal in single-metal systems, a decrease in thiol content was observed for Cu(II), and there is a tendency for an increase in thiol content under Ni(II) action. The increase of the total thiols content becomes especially pronounced at Ni(II) concentrations of 50 mg/L; Cu(II) at this concentration continues to keep the total thiols content at a level lower than the control level. Zn(II) at both concentrations did not influence significantly the total thiol content.

An interesting synergistic effect is observed at the joint action of the studied metals (Figure 6B): the effect of Cu(II) in the case of Cu/Zn and Cu/Ni/Zn systems consists in a decrease of the total thiol content in the cells, which means that Cu(II) effect dominates in these combinations. However, the effect of Cu(II) to diminish the thiol content was neutralized by Ni(II) in the Cu/Ni system. The particular efficiency of Ni(II) in thiol homeostasis was observed in the Cu/Ni system. As for the Ni/Zn system, the Ni(II) effect was so pronounced that it could be considered dominant. Despite the tendency to reduce the level of thiols under such a strong agent as Cu(II), the system obeys the tendency of Ni(II), which is expressed in an increase in the thiol levels in comparison with the control.

## 4. Discussion

*Shewanella* strains have been isolated from different environments, such as coastal sea sediments [41], marine and freshwater environments, inhabitants [42], heavy-metal-enriched environments [43]*,* and the present study from radionuclide-contaminated areas [17]. Members of the genus *Shewanella* are heterotrophic, facultative aerobes that use a large number of organic and inorganic substances as energy sources. Many of these organisms can respire a wide assortment of naturally occurring soluble and insoluble metal/metalloid oxides [44].

Bacteria in natural conditions are faced with various metal-related stresses and can develop very exquisite mechanisms of essential and toxic element uptake, exclusion, and/or compartmentalization. *Shewanella* strains encode an array of proteins that allow them to resist heavy metal effects [43,44]. Data obtained in the present study on the annotated genes for *S. xiamenensis* DCB2-1, which allow revealing its resistance toward Cu(II), Zn(II), and Ni(II), confirm this fact. Considering *S. xiamenensis* DCB2-1 survival rate, it was shown that Zn(II) and Ni(II) in single-metal systems did not provoke particular toxicity, even at the highest concentration of 200 mg/L. The cell decrease was within one order of magnitude in all analyzed systems, except the Cu(II) system, in which complete cell destruction at a metal concentration of 200 mg/L was observed (Figure 1).

During metal accumulation in the biomass, some co-occurrence had been observed between the cell viability and the efficiency of the uptake or change of metal levels in the biomass. In the case of the Zn(II) system, an increase in the zinc concentration in the growth medium increased the efficiency of the uptake (94%), and consequently its content in biomass (Table 1). The sharp rise in zinc content in biomass (starting at a concentration of 50 mg/L) coincided with an insignificant decrease in cell survival. In the case of Ni(II), with an increase in metal concentration in the medium, a significant decrease in the efficiency of uptake (from 21.3% at 5 mg/L to 9.3% at 200 mg/L) was observed, which corresponded with high cell survival. As for Cu(II), the metal content in the biomass and the uptake efficiency with increasing metal concentration in the medium were commensurate with Ni(II); however, an increase in the copper uptake starting at 75 mg/L Cu(II) coincided with a decrease in cell survival.

Severe control of the homeostasis of metal ions is critical for all forms of life. Zn(II) is buffered in the nanomolar [45] to picomolar [46] range. Bacteria have elaborated both import and export mechanisms to strictly control the level of zinc accessible for zinc-requiring proteins and enzymes involved in metabolic processes in the cell [47,48]. In the case of *S. xiamenensis* DSB2-1, there is the ZntR transporter (ONJFFADL_00816); transcription factors (ONJFFADL_01264 and ONJFFADL_03080), which form zinc resistance; CzcA and CzcB, which regulate the concentrations of Cd, Zn, and Co (ONJFFADL_02598 and ONJFFADL_02597), etc. (Supplementary Materials). Post-efflux mechanisms for zinc exist, which restrict the secondary entry of effluxed zinc ions in cells by precipitation, attachment to a protein, or periplasmic storage [49].

Copper is an important cofactor for oxidative stress-related enzymes including catalase, superoxide dismutase, peroxidase, cytochrome c oxidases, ferroxidases, monoamine oxidase, and dopamine β-monooxygenase [50,51]. Bacterial copper homeostasis differs significantly from zinc homeostasis due to the existence of a chemical equilibrium between cupric Cu(II) and cuprous Cu(I) ions. Cu(I) is present in cells at attomolar levels, and usually these ions are readily complexed by various biological ligands [52,53]. Cu(II) is considered a relatively safe species and is biologically more inert than Cu(I) [54]. Copper can enter the periplasm by crossing the bacterial outer membrane with the aid of Cu(I)/Cu(II) porin ATPases [55]. Only a few Cu transporters have been annotated to date, and all of them are characterized by different mechanisms [56]. Cu(I) exporting ATPase (https://www.ncbi.nlm.nih.gov/protein/TVL37167.1, accessed on 30 June 2022); CusA/CzcA family heavy metal efflux RND transporter (https://www.ncbi.nlm.nih.gov/gene/72485104, accessed on 30 June 2022); Cu(I)-sensitive transcription regulators (ONJFFADL_02162, ONJFFADL_02459) for activation of the copper efflux system; and cusB subunit (ONJFFADL_02433) of the periplasmic outflow adapter RND are present in genomes, including annotated genes of the *S. xiamenensis* DCB2-1.

Nickel is also a well-known cofactor of several key microbial enzymes, including urease, [NiFe] hydrogenase, Ni-superoxide dismutase, carbon monoxide dehydrogenase, acetyl CoA synthase/decarbonylase, and methyl coenzyme M reductase, etc. [57]. Metal transporters and nickel efflux, as well as other detoxification mechanisms developed to reduce its toxicity, provide nickel homeostasis in microorganisms [58]. In the case of *S. xiamenensis* DSB2-1, Ni(II) resistance can be regulated by ATP-dependent transporters (ONJFFADL_03077, ONJFFADL_03076, and ONJFFADL_01609), TonB-dependent siderophore receptor (ONJFFADL_01301), etc. (Supplementary Materials). Analysis of the *S. xiamenensis* DCB2-1 genome shows that for zinc, the bacteria have a bunch of transporter genes; for copper, mainly genes for copper-removing proteins are present; and the nickel-resistant genes in *S. xiamenensis* DCB2-1 occupy the third place in the row of metal resistance genes of this bacterium, which also include transporters and stress-resistant genes (Figure 3). Obviously, this can explain the character of single-metal accumulation and *S. xiamenensis* DCB2-1 survival observed in the present study, namely, a high accumulating capacity for Zn(II) and a low accumulating capacity for Cu(II) and Ni(II).

The multi-metal systems are used to model real metal-contaminated environments. The combination Cu/Ni/Zn = 2/2/10 mg/L is a combination of the studied metals characteristic of real effluents from the electroplating industry. In the present study, the influence of multi-metal systems, containing different combinations of three metals, on the survival and accumulating capacity of the bacterium *S. xiamenensis* DCB2-1 was estimated. It should be emphasized that the combination Cu/Ni/Zn = 20/20/20 mg/L is not toxic for *S. xiamenensis* DCB2-1, and the bacterium was able to survive under these conditions. The discussion of the accumulation of metals in multi-metal systems will focus on the assignment of synergy among the interactions of different metals in the studied combinations. Synergy is most often defined in relation to pharmacology and medicine [59]. However, synergistic effects have been observed in chemical mixtures in toxicological and environmental studies as well. The heavy metals simultaneous action can lead to positive or negative synergistic responses in metallophylic bacteria. In the case of a positive correlation, accumulation of some metals facilitates uptake of other metals by bacteria. For the negative correlation, accumulation of some metals prevents/decreases uptake of other metals. It was demonstrated that Zn blocks Mn uptake in *Streptoccocus pneumoniae* [60], and Zn excess perturbs the Fe and Cu homeostasis in *E. coli*, resulting in an increased demand for Fe supply and a reduced tolerance to Cu toxicity [61,62]. Mn accumulation can prevent the accumulation of other metals by intestinal microbiota. At the same time, Fe accumulation facilitates Co, Pb, and Zn uptake [63].

To evaluate and examine the synergistic action of the studied metals, their content in biomass in multi-metal systems was compared with the content in single-metal systems at the same concentrations (Table 1 and Table 2). In multi-metal systems at metal concentrations of 20 mg/L, the uptake picture differed from that obtained for Cu(II) and Ni(II) systems. Thus, Cu content increased 40–50 fold in Cu/Zn and Cu/Ni combinations; Ni content increased six fold in the combinations Ni/Zn and Cu/Ni; and Zn content did not change in Ni/Zn and Cu/Zn combinations compared to single-metal systems. In the ternary metal combination Cu/Ni/Zn, the content of Cu increased 40–50 fold as in Cu/Zn and Cu/Ni combinations, the Ni content increased six fold as in the combinations Ni/Zn and Cu/Ni, and the Zn content did not change and was on the level of Ni/Zn and Cu/Zn combinations. In multi-metal systems, at a concentration of 50 mg/L, the pattern differed from that observed for a concentration of 20 mg/L: Cu content increased 10–20 fold in the Cu/Zn and Cu/Ni systems; Ni content increased seven fold in the Ni/Zn and Cu/Ni systems; Zn content did not change in the Ni/Zn system and decreased 10 fold in the Cu/Zn system. Therefore, in the Cu/Ni system at metal concentrations of 20 and 50 mg/L, positive synergism has been observed for both metals; in the Ni/Zn system at concentrations of 20 and 50 mg/L, Zn promoted the uptake of Ni and can be considered a positive synergist for Ni; in the Cu/Zn system at metal concentrations of 20 mg/L, Zn was again a positive synergist for Cu; however, at a metal concentration of 50 mg/L, Cu acted as a negative synergist for Zn, reducing its uptake. In the ternary system, at metal concentrations of 20 mg/L, Zn was a positive synergist for both Ni and Cu.

The action of single and multiple metals on the metabolism of *S. xiamenensis* DCB2-1 regarding the antioxidant enzymes was evaluated since studied metals directly or indirectly participate in ROS production.

The ability of copper to cycle between an oxidized and reduced state is used by cuproenzymes, which are involved in redox reactions [50,51]. Meanwhile, this particularity makes copper potentially toxic, since the transitions between copper states can lead to the generation of superoxide and hydroxyl radicals [8,50,51]. The generation of ROS is regarded as the main mechanism of copper ion toxicity [64]. Zinc combats the catalytic properties of the redox-active transition metals, such as iron and copper, leading to the formation of OH radicals from H_2_O_2_ and superoxide via the Fenton reaction [65]. Nickel, in comparison with iron or copper, is a rather poor generator of ROS [66]. However, a variety of accessory ligands that bind nickel can facilitate its ability to produce ROS. Thus, nickel binding to cysteine, histidine, glutathione [67], small peptides [68], and amine-containing compounds [69] can speed up the rate of oxidative damage provoked by metal. Nickel can lead to protein oxidation, forming Tyr-Tyr crosslinks; it stimulates lipid oxidation in the presence of thiols or in combination with iron and copper. Nickel uses a variety of oxidizing agents in these reactions, including oxygen, superoxide, hydrogen peroxide, and organic hydroperoxides [69].

Catalase is one of the major enzymes playing an important role in bacteria’s protection against metal toxicity caused by oxidative stress. The catalase activity of *S. xiamenensis* DCB2-1 was inhibited in single and multi-metal combinations containing Cu(II) at a concentration of 20 mg/L (Figure 4). It is suggested that further increases in Cu(II) concentration in the multiple systems may lead to cell destruction because the catalase activity is already inhibited at a concentration of 20 mg/L. Such an inhibitory effect of copper on soil catalase activity was observed at amaranth cultivation in the soil supplemented with copper in a wide range of concentrations. The rate of catalase activity decreases with an increase in copper concentration [70]. However, the catalase activity of *S. xiamenensis* DCB2-1 increased significantly in Cu(II) and Zn(II) systems containing metals at concentrations of 50 mg/L and in the Ni(II) system at metal concentrations of 20 and 50 mg/L. Catalase in these conditions is exploited as an enzyme for opposing oxidative stress.

SOD is another major antioxidant enzyme. There are different isoforms of SOD in bacteria: Cu, ZnSOD, MnSOD, NiSOD, FeSOD, and FeMnSOD. It was identified the type of SOD in *S. xiamenensis* DCB2-1 as FeSOD isoform (protein ONJFFADL_03232, Supplementary Materials), the same type as in the case of the strain *S. xiamenensis* CQ-Y1 (sodB Gene ID: 72482664, https://www.ncbi.nlm.nih.gov/gene/72482664, accessed on 12 July 2022). The in-gel assay (Figure 5) demonstrated one remarkably strong visible line corresponding to SOD activity. SOD proved to be an active enzyme for combating oxidative stress in the following conditions: at metal concentrations of 20 mg/L in Cu(II) and Zn(II) systems; at metal concentrations of 50 mg/L in Cu(II) and Ni(II) systems; and in all multi-metal systems containing Cu(II). Based on these data, a conclusion could be drawn about the particular relevance of SOD activity to Cu(II) action, either alone or in combination with Zn(II) and Ni(II). Only in the Ni/Zn system at a metal concentration of 20 mg/L was SOD activity inhibited, and as previously discussed, Ni(II) uptake by biomass in these conditions increased sixfold. Ni(II) reveals the property to inhibit iron-containing enzymes. For example, E. coli needed increased iron uptake during high nickel accumulation in order to prevent the disincorporation of nickel into iron metalloenzymes. It was reported that many iron-containing enzymes can be inhibited by nickel, which has the ability to replace iron in the enzyme active center [71].

The nucleophilic sulfur containing thiol groups is represented by cysteine and highly abundant low molecular thiols, which have a wide range of functions in the cell. Cysteine is often found as a conserved residue at the functional sites of proteins. Free thiols, which are important components of the thiol/disulfide buffer, are used as metal chelators. They play a critical role in the homeostasis of the cellular redox state and in the detoxification of ROS [72,73]. It is generally favorable to increase the availability of specific antioxidants under conditions of oxidative stress. Cells have developed a number of mechanisms to promote the increase of thiol levels. According to Figure 6, Ni(II) single action and the combination Ni/Zn increased the total thiols, which can be associated with increased tolerance to oxidative stresses and correlated with high survival in these systems (Figure 1 and Figure 2).

*Shewanella* species belonging to dissimilatory metal bacteria can attract great interest for bioremediation. *S. xiamenensis* DCB2-1, under the actions of different combinations of metals for 24 h, may exist in the form of live and dead biomass, depending on metal concentrations of 20 mg/L and 200 mg/L, respectively. Metal uptake efficiencies have been compared for these two biological states (Table 2). In the Cu/Ni system, *S. xiamenensis* DCB2-1 accumulated 50.4% copper as live biomass, which is comparable with the removal efficiency of dead biomass of 43.2%. Nickel was removed two times better from dead biomass (21.8%) compared with live biomass (11%). In the combination Cu/Zn, live biomass (48.2%) accumulated three times more copper than dead biomass (14.9%), and zinc was accumulated two times better in dead biomass (19.6%) than in live biomass (9.1%). In the combination Cu/Ni/Zn, live biomass accumulated three times more copper (86.6%) than dead biomass (28%); zinc accumulation in dead biomass of 78.6% was six times higher than in live biomass (12.7%). Therefore, the bacterial capacity for metal removal from the environment depends on the metal combinations and the biological state of the biomass. Thus, the biological state of the biomass should be taken into consideration for the recommendation of a bacterial strain as the biosorbent.

## 5. Conclusions

Bacterial resistance to metals depends on their sensitivity to metal ions, as defined by the metal uptake systems, the role, and interactions of the metal in the normal metabolism of the cell. Genetic and biochemical bases determine these factors. The data presented in the study demonstrate the heterogeneous nature of the bacterial strain *S. xiamenensis* DCB2-1’s adaptation to stress caused by Cu(II), Zn(II), and Ni(II) single and multi-metal actions. The order of toxicity of the metals to the bacterium was as follows: Cu > Zn > Ni. In the single-metal system, copper accumulation in cell culture was low at first, gradually increasing with its concentration. A considerable amount of copper was accumulated in multi-metal systems such as Cu/Ni, Cu/Zn, and Cu/Ni/Zn, while zinc and nickel acted as positive synergists. Copper accumulation in single and multi-metal systems caused oxidative stress, resulting in a decrease in the total amount of thiols in the biomass. Similar to Cu(II), low Ni(II) accumulation in the single-metal system was followed by a steadily increasing amount of its content in the cells with the increase in metal concentration in solution. The content of nickel in bacteria rose in the Ni/Zn and Cu/Ni systems, where zinc and copper acted as positive synergists. The fact that the concentration of total thiols was higher than normal in the case of single and mixed nickel exposure was a sign of the oxidative state. Compared to copper and nickel, zinc removal from the medium was greater in the single-metal system, and it did not induce factual toxicity in single-metal systems at the studied concentration range. At the same time, nickel and copper in multi-metal systems inhibit zinc accumulation, acting as negative synergists. Zinc accumulation altered the oxidative state of the bacteria, as evidenced by the total thiol content. The antioxidant defense system responded differently to metal-induced redox homeostasis imbalances. Copper’s harmful action in multi-metal systems was reduced by SOD and catalase. Under zinc action, SOD protected cells by increasing its activity, whereas catalase was not actively involved. SOD did not actively participate in the nickel stress response, while catalase increased its activity.

## Figures and Tables

**Table 1:** The content of metal accumulated in biomass and the efficiency of metal uptake from single-metal solutions.

System	Concentration in Solution, mg/L	Efficiency of Uptake, %	Content in Biomass, mg/g
Cu(II)	5	19	2.3
	20	8.8	4.6
	50	5.9	6.0
	75	10.3	17.7
	100	16.1	38.3
Ni(II)	5	21.3	4.02
	20	14.0	9.7
	50	14.6	21.3
	75	9.6	20.4
	100	9.5	26.4
	200	9.3	30.1
Zn(II)	5	51	6.2
	20	93	49
	50	90	103
	75	96	168
	100	92	229
	200	94	288

**Table 2:** The content of metal accumulated in biomass and the efficiency of metal uptake from multi-metal solutions.

System	Metal	Efficiency of Uptake, %	Content in Biomass, mg/g
20 mg/L for each metal			
Cu/Ni	Cu	50.4	226.6
	Ni	11.0	61.2
Ni/Zn	Ni	11.8	63.7
	Zn	11.5	43.6
Cu/Zn	Cu	48.2	197.8
	Zn	9.1	33.1
Cu/Ni/Zn	Cu	86.6	254.7
	Ni	11.0	41.0
	Zn	12.7	32.9
50 mg/L for each metal			
Cu/Ni	Cu	13.4	134.9
	Ni	12.1	154.2
Ni/Zn	Ni	13.1	164.9
	Zn	12.9	111.5
Cu/Zn	Cu	7.9	80.3
	Zn	1.0	8.6
Cu/Ni/Zn	Cu	16.1	113.7
	Ni	12.6	115.4
	Zn	10.7	65.6
200 mg/L for each metal			
Cu/Ni	Cu	43.2	186.4
	Ni	21.8	142.5
Ni/Zn	Ni	20.7	89.3
	Zn	78.4	326.7
Cu/Zn	Cu	14.9	48.2
	Zn	19.6	79.2
Cu/Ni/Zn	Cu	28.0	92.9
	Ni	21.3	93.2
	Zn	78.6	329.2

## Data Availability

Not applicable.

## Supplementary Materials

The following supporting information can be downloaded at: https://www.mdpi.com/article/10.3390/toxics11040304/s1. Table S1: Shewanella xiamenenis DCB 2-1 genes.

## References

[1] Haynes W.M. (2015). CRC Handbook of Chemistry and Physics.

[2] Entwistle J.A., Hursthouse A.S., Marinho Reis P.A., Stewart A.G. (2019). Metalliferous Mine Dust: Human Health Impacts and the Potential Determinants of Disease in Mining Communities. Curr. Pollut. Rep..

[3] Tchounwou P.B., Yedjou C.G., Patlolla A.K., Sutton D.J., Luch A. (2012). Heavy Metal Toxicity and the Environment. Molecular, Clinical and Environmental Toxicology. Experientia Supplementum.

[4] Landrigan P.J. (2018). The Lancet Commission on pollution and health. Lancet.

[5] Burakov A.E., Galunin E.V., Burakova I.V., Kucherova A.E., Agarwal S., Tkachev A.G., Gupta V.K. (2018). Adsorption of heavy metals on conventional and nanostructured materials for wastewater treatment purposes: A review. Ecotoxicol. Environ. Saf..

[6] Keiblinger K.M., Schneider M., Gorfer M., Paumann M., Deltedesco E., Berger H., Jöchlinger L., Mentler A., Zechmeister-Boltenstern S., Soja G. (2018). Assessment of Cu applications in two contrasting soils—Effects on soil microbial activity and the fungal community structure. Ecotoxicology.

[7] Sisodia N.S. (2020). Effect of Heavy Metal Toxicity on Environment and Health. Int. J. Res. Appl. Sci. Biotechnol..

[8] Tchounwou P., Newsome C., Williams J., Glass K., Collery P., Maymard I., Thephanides T., Khassanova L., Collery T. (2008). Copper-induced cytotoxicity and transcriptional activation of stress genes in human liver carcinoma (HepG2) cells. Metal Ions in Biology and Medicine.

[9] Rouch D.A., Lee B.T.O., Morby A.P. (1995). Understanding cellular responses to toxic agents: A model for mechanism-choice in bacterial metal resistance. J. Ind. Microbiol..

[10] Mammari N., Lamouroux E., Boudier A., Duval R.E. (2022). Current knowledge on the oxidative-stress-mediated antimicrobial properties of metal-based nanoparticles. Microorganisms.

[11] Khanna K., Kohli S.K., Handa N., Kaur H., Ohri P., Bhardwaj R., Yousaf B., Rinklebe J., Ahmad P. (2021). Enthralling the impact of engineered nanoparticles on soil microbiome: A concentric approach towards environmental risks and cogitation. Ecotoxicol. Environ. Saf..

[12] Wang L., Hu C., Shao L. (2017). The antimicrobial activity of nanoparticles: Present situation and prospects for the future. Int. J. Nanomed..

[13] Singh J., Vishwakarma K., Ramawat N., Rai P., Singh V.K., Mishra R.K., Kumar V., Tripathi D.K., Sharma S. (2019). Nanomaterials and microbes’ interactions: A contemporary overview. 3 Biotech.

[14] Seixas A.F., Quendera A.P., Sousa J.P., Silva A.F.Q., Arraiano C.M., Andrade J.M. (2022). Bacterial Response to Oxidative Stress and RNA Oxidation. Front. Genet..

[15] Borisov V.B., Siletsky S.A., Nastasi M.R., Forte E. (2021). ROS defense systems and terminal oxidases in bacteria. Antioxidants.

[16] Asatiani N., Kartvelishvili T., Sapojnikova N., Abuladze M., Asanishvili L., Osepashvili M. (2018). Effect of the Simultaneous Action of Zinc and Chromium on *Arthrobacter* spp. Water. Air. Soil Pollut..

[17] Grouzdev D.S., Safonov A.V., Babich T.L., Tourova T.P., Krutkina M.S., Nazina T.N. (2018). Draft genome sequence of a dissimilatory U(VI)- reducing bacterium, *Shewanella xiamenensis* strain DCB2-1, isolated from nitrate- and radionuclide-contaminated groundwater in Russia. Genome Announc..

[18] Zinicovscaia I., Safonov A., Ostalkevich S., Gundorina S., Nekhoroshkov P., Grozdov D. (2019). Metal ions removal from different type of industrial effluents using Spirulina platensis biomass. Int. J. Phytoremediation.

[19] Zinicovscaia I., Yushin N., Grozdov D., Safonov A., Ostovnaya T., Boldyrev K., Kryuchkov D., Popova N. (2021). Bio-zeolite use for metal removal from copper-containing synthetic effluents. J. Environ. Health Sci. Eng..

[20] Zinicovscaia I., Yushin N., Grozdov D., Vergel K., Popova N., Artemiev G., Safonov A. (2020). Metal removal from nickel-containing effluents using mineral–organic hybrid adsorbent. Materials.

[21] Zinicovscaia I., Yushin N., Grozdov D., Abdusamadzoda D., Safonov A., Rodlovskaya E. (2021). Zinc-containing effluent treatment using Shewanella xiamenensis biofilm formed on zeolite. Materials.

[22] Herigstad B., Hamilton M., Heersink J. (2001). How to optimize the drop plate method for enumerating bacteria. J. Microbiol. Methods.

[23] Hoben H.J., Somasegaran P. (1982). Comparison of the pour, spread, and drop plate methods for enumeration of *Rhizobium* spp. in inoculants made from presterilized peat. Appl. Environ. Microbiol..

[24] Laemmli U.K. (1970). Cleavage of structural proteins during the assembly of the head of bacteriophage T4. Nature.

[25] Steinman H.M. (1985). Bacteriocuprein superoxide dismutases in pseudomonads. J. Bacteriol..

[26] Hawkins C.L., Morgan P.E., Davies M.J. (2009). Quantification of protein modification by oxidants. Free Radic. Biol. Med..

[27] Shingler V. (1996). Signal sensing by σ54-dependent regulators: Derepression as a control mechanism. Mol. Microbiol..

[28] Sánchez-Sutil M.C., Pérez J., Gómez-Santos N., Shimkets L.J., Moraleda-Muñoz A., Muñoz-Dorado J. (2013). The *Myxococcus xanthus* Two-Component System CorSR Regulates Expression of a Gene Cluster Involved in Maintaining Copper Tolerance during Growth and Development. PLoS ONE.

[29] Hiniker A., Collet J.F., Bardwell J.C.A. (2005). Copper stress causes an in vivo requirement for the Escherichia coli disulfide isomerase DsbC*. J. Biol. Chem..

[30] Shi W., Zhang B., Jiang Y., Liu C., Zhou W., Chen M., Yang Y., Hu Y., Liu B. (2021). Structural basis of copper-efflux-regulator-dependent transcription activation. iScience.

[31] Stoyanov J.V., Hobman J.L., Brown N.L. (2001). CueR (Ybbl) of *Escherichia coli* is a MerR family regulator controlling expression of the copper exporter CopA. Mol. Microbiol..

[32] Changela A., Chen K., Xue Y., Holschen J., Outten C.E., O’Halloran T.V., Mondragón A. (2003). Molecular basis of metal-ion selectivity and zeptomolar sensitivity by CueR. Science.

[33] Munson G.P., Lam D.L., Outten F.W., O’Halloran T.V. (2000). Identification of a copper-responsive two-component system on the chromosome of *Escherichia coli* K-12. J. Bacteriol..

[34] Franke S., Grass G., Rensing C., Nies D.H. (2003). Molecular analysis of the copper-transporting efflux system CusCFBA of *Escherichia coli*. J. Bacteriol..

[35] Brocklehurst K.R., Hobman J.L., Lawley B., Blank L., Marshall S.J., Brown N.L., Morby A.P. (1999). ZntR is a Zn(II)-responsive MerR-like transcriptional regulator of zntA in *Escherichia coli*. Mol. Microbiol..

[36] Jovanovic G., Dworkin J., Model P. (1997). Autogenous control of PspF, a constitutively active enhancer-binding protein of *Escherichia coli*. J. Bacteriol..

[37] Goldberg M., Pribyl T., Juhnke S., Nies D.H. (1999). Energetics and topology of CzcA, a cation/proton antiporter of the resistance-nodulation-cell division protein family. J. Biol. Chem..

[38] Albareda M., Rodrigue A., Brito B., Ruiz-Argüeso T., Imperial J., Mandrand-Berthelot M.A., Palacios J. (2015). *Rhizobium leguminosarum* HupE is a highly-specific diffusion facilitator for nickel uptake. Metallomics.

[39] Samantarrai D., Sagar A.L., Gudla R., Siddavattam D. (2020). TonB-Dependent Transporters in Sphingomonads: Unraveling Their Distribution and Function in Environmental Adaptation. Microorganisms.

[40] Eitinger T., Culotta V., Scott R.A. (2013). Nickel, Cobalt Transport in Prokaryotes. Metals and Cells.

[41] Huang J., Sun B., Zhang X. (2010). *Shewanella xiamenensis* sp. nov., isolated from coastal sea sediment. Int. J. Syst. Evol. Microbiol..

[42] Huang Y.T., Cheng J.F., Wu Z.Y., Tung K.C., Chen Y.J., Hong Y.K., Chen S.Y., Liu P.Y. (2019). Genomic and phylogenetic characterization of *Shewanella xiamenensis* isolated from giant grouper (*Epinephelus lanceolatus*) in Taiwan. Zoonoses Public Health.

[43] Harris H.W., Sánchez-Andrea I., McLean J.S., Salas E.C., Tran W., El-Naggar M.Y., Nealson K.H. (2018). Redox sensing within the genus *Shewanella*. Front. Microbiol..

[44] Barchinger S.E., Pirbadian S., Sambles C., Baker C.S., Leung K.M., Burroughs N.J., El-Naggar M.Y., Golbeck J.H. (2016). Regulation of gene expression in *Shewanella oneidensis* MR-1 during electron acceptor limitation and bacterial nanowire formation. Appl. Environ. Microbiol..

[45] Wang D., Hosteen O., Fierke C.A. (2012). ZntR-mediated transcription of zntA responds to nanomolar intracellular free zinc. J. Inorg. Biochem..

[46] Reyes-Caballero H., Campanello G.C., Giedroc D.P. (2011). Metalloregulatory proteins: Metal selectivity and allosteric switching. Biophys. Chem..

[47] Braymer J.J., Giedroc D.P. (2014). Recent developments in copper and zinc homeostasis in bacterial pathogens. Curr. Opin. Chem. Biol..

[48] Outten C.E., O’Halloran T.V. (2001). Femtomolar sensitivity of metalloregulatory proteins controlling zinc homeostasis. Science..

[49] Nanda M., Kumar V., Sharma D.K. (2019). Multimetal tolerance mechanisms in bacteria: The resistance strategies acquired by bacteria that can be exploited to ‘clean-up’ heavy metal contaminants from water. Aquat. Toxicol..

[50] Stern B.R. (2010). Essentiality and toxicity in copper health risk assessment: Overview, update and regulatory considerations. J. Toxicol. Environ. Health A.

[51] Harvey L.J., McArdle H.J. (2008). Biomarkers of copper status: A brief update. Br. J. Nutr..

[52] Barwinska-Sendra A., Waldron K.J. (2017). The Role of Intermetal Competition and Mis-Metalation in Metal Toxicity. Adv. Microb. Physiol..

[53] Dupont C.L., Grass G., Rensing C. (2011). Copper toxicity and the origin of bacterial resistance—New insights and applications. Metallomics.

[54] Stewart L.J., Thaqi D., Kobe B., McEwan A.G., Waldron K.J., Djoko K.Y. (2019). Handling of nutrient copper in the bacterial envelope. Metallomics.

[55] Raimunda D., González-Guerrero M., Leeber B.W., Argüello J.M. (2011). The transport mechanism of bacterial Cu+-ATPases: Distinct efflux rates adapted to different function. BioMetals.

[56] Bhamidimarri S.P., Young T.R., Shanmugam M., Soderholm S., Basle A., Bumann D., Van Den Berg B. (2021). Acquisition of ionic copper by the bacterial outer membrane protein OprC through a novel binding site. PLoS Biol..

[57] Mulrooney S.B., Hausinger R.P. (2003). Nickel uptake and utilization by microorganisms. FEMS Microbiol. Rev..

[58] MacOmber L., Hausinger R.P. (2011). Mechanisms of nickel toxicity in microorganisms. Metallomics.

[59] Roell K.R., Reif D.M., Motsinger-Reif A.A. (2017). An introduction to terminology and methodology of chemical synergy—Perspectives from across disciplines. Front. Pharmac..

[60] McDevitt C.A., Ogunniyi A.D., Valkov E., Lawrence M.C., Kobe B., McEwan A.G., Paton J.C. (2011). A molecular mechanism for bacterial susceptibility to Zinc. PLoS Pathog..

[61] Casanova-Hampton K., Carey A., Kassam S., Garner A., Donati G.L., Thangamani S., Subashchandrabose S. (2021). A genome-wide screen reveals the involvement of enterobactin-mediated iron acquisition in *Escherichia coli* survival during copper stress. Metallomics.

[62] Xu Z., Wang P., Wang H., Yu Z.H., Au-Yeung H.Y., Hirayama T., Sun H., Yan A. (2019). Zinc excess increases cellular demand for iron and decreases tolerance to copper in *Escherichia coli*. J. Biol. Chem..

[63] Sizentsov A., Sizentsov Y., Kvan O., Salnikova E., Salnikova V. (2019). A study on heavy metal sorption properties of intestinal microbiota in vitro. E3S Web Conf..

[64] Halliwell B., Gutteridge J.M.C., Halliwell B., Gutteridge J.M.C. (2015). Redox chemistry: The essentials. Free Radicals in Biology and Medicine.

[65] Korbashi P., Katzhandler J., Saltman P., Chevion M. (1989). Zinc protects *Escherichia coli* against copper-mediated paraquat-induced damage. J. Biol. Chem..

[66] Valko M., Morris H., Cronin M. (2005). Metals, toxicity and oxidative stress. Curr. Med. Chem..

[67] Joshi S., Husain M.M., Chandra R., Hasan S.K., Srivastava R.C. (2005). Hydroxyl radical formation resulting from the interaction of nickel complexes of L-histidine, glutathione or L-cysteine and hydrogen peroxide. Hum. Exp. Toxicol..

[68] Shi X., Dalal N.S., Kasprzak K.S. (1992). Generation of free radicals from lipid hydroperoxides by Ni2+ in the presence of oligopeptides. Arch. Biochem. Biophys..

[69] Shi X., Kasprzak K.S., Dalal N.S. (1993). Generation of Free radicals in reactions of Ni(II)-thiol complexes with molecular oxygen and model lipid hydroperoxides. J. Inorg. Biochem..

[70] Skwaryło-Bednarz B., Krzepiłko A., Brodowska M.S., Brodowski R., Ziemińska-Smyk M., Onuch J., Gradziuk B. (2018). The impact of copper on catalase activity and antioxidant properties of soil under amaranth cultivation. J. Elem..

[71] Wang S., Wu Y., Outten F.W. (2011). Fur and the novel regulator YqjI control transcription of the ferric reductase gene yqjH in *Escherichia coli*. J. Bacter..

[72] Ulrich K., Jakob U. (2019). The role of thiols in antioxidant systems. Free Radic. Biol. Med..

[73] Deneke S.M. (2001). Thiol-based antioxidants. Curr. Top. Cell. Regul..

